# Influence of Nutrient Deprivation on the Antioxidant Capacity and Chemical Profile of Two Diatoms from Genus *Chaetoceros*

**DOI:** 10.3390/md22020096

**Published:** 2024-02-19

**Authors:** Roberta Frleta Matas, Sanja Radman, Martina Čagalj, Vida Šimat

**Affiliations:** 1 Center of Excellence for Science and Technology-Integration of Mediterranean Region (STIM), Faculty of Science, University of Split, Rudera Boškovića 35, 21000 Split, Croatia; roberta@stim.unist.hr; 2 Department of Food Technology and Biotechnology, Faculty of Chemistry and Technology, University of Split, Ruđera Boškovića 35, 21000 Split, Croatia; 3 University Department of Marine Studies, University of Split, Rudera Boškovića 37, 21000 Split, Croatia; mcagalj@unist.hr

**Keywords:** phytoplankton, DPPH, ORAC, UHPLC-ESI-HRMS, fatty acid amides, pigments

## Abstract

The limited availability of phosphate, nitrogen and silicon in the growth media affects the growth, cellular processes, and metabolism of diatoms. Silicon deficiency primarily affects diatom morphology, while phosphate deficiency reduces the production of nucleic acids and phospholipids. Differences in pigment and protein composition are mainly due to nitrogen deficiency. In this study, *Chaetoceros socialis* and *Chaetoceros costatus* were cultured under phosphate, nitrogen, and silicon deprivation conditions. The diatom biomass was collected during the stationary growth phase and extracted with 70% ethanol under ultrasonication. The chemical profiles of the extracts were analyzed by high-performance liquid chromatography with high-resolution mass spectrometry with electrospray ionisation (UHPLC-ESI-HRMS), while the antioxidant capacity was determined by 2,2-diphenyl-1-picrylhydrazyl (DPPH) radical scavenging and oxygen radical absorbance capacity (ORAC) assays. Pigments, fatty acids, sterols, and derivatives were detected in both species. The total phenolic content in the extracts ranged from 46.25 ± 1.08 to 89.38 ± 6.21 mg of gallic acid equivalent (GAE)/L and from 29.58 ± 1.08 to 54.17 ± 1.18 mg GAE/L. for *C. costatus* and *C. socialis*, respectively. Antioxidant activity was higher in *C. costatus* extracts, especially those obtained from nitrogen-deprived media. The results of this study contribute to the existing knowledge and the ongoing efforts to overcome application and commercialization barriers of microalgae for wide-ranging potential in different industries.

## 1. Introduction

Within the marine ecosystem, microalgae constitute a significant group of photosynthetic microorganisms, playing a vital role in the marine environment. Marine microalgae produce about 50% of atmospheric oxygen and present the most important food source in marine ecosystems [1]. The most abundant group of marine microalgae are diatoms, which are responsible for over 40% of the total primary production of the oceans [2]. As diatoms are widely distributed in various marine environments, from polar to tropical regions, they are highly adaptable to different abiotic parameters. Temperature, salinity, pH, light intensity, CO_2_ concentration and especially the availability of nutrients are parameters that have a great influence on the growth and production of the metabolites of diatoms [3,4,5].

Among the macronutrients that have the greatest influence on the growth and metabolism of diatoms, silicon (Si), nitrogen (N) and phosphorus (P) are of primary importance. Si plays a role in the marine environment by controlling primary productivity [6]. In diatom growth, silicon requirements are related to the process of building a cell wall (frustule) but are also indirectly involved and important for cellular metabolic processes such as DNA replication and cell division [7,8]. On the other hand, N is an essential nutrient required for the biosynthesis of many molecules, including amino acids, nucleic acids, lipids, and some sugars, but most of the assimilated nitrogen is used for the synthesis of proteins and nucleic acids [7]. For the production of chemical energy in the form of NADPH and ATP, diatoms use P. Phosphorus is also an important part of diatom cell membranes, where it is found in the form of phospholipids and is a component of nucleic acids (DNA and RNA) [9].

A higher concentration of these macronutrients in the growing media usually leads to an increased growth rate, while their limitation or deficiency affects the synthesis of compounds such as phenols, pigments, and fatty acids [7,10]. Nutrient deprivation with N, P and Si could be a stress trigger needed to increase the metabolic production and accumulation of various bioactive compounds in diatom cells [9,10,11,12]. Previous studies have shown that the species *Chaetoceros muelleri* experiences the greatest physiological stress under nitrogen deprivation [3,13]. In addition to a higher proportion of lipids, a decrease in the content of *n*-3 polyunsaturated fatty acids (PUFA) was also observed, while the proportion of monounsaturated fatty acids (MUFA) increased in these conditions [3]. The same results of lipid increase were observed in the species *Phaeodactylum tricornutum*, where a decrease in phenolic compounds and pigments was also observed during cultivation under N-deprivation compared to standard culture conditions [10]. In diatoms, the nutrient deficiency of Si and P has the same effects on metabolic processes [11,14]. Inducing stress by complete nutrient deprivation acts in the same way as a limitation of particular key nutrients; however, the metabolic response is species-dependent [14].

Therefore, the aim of this study was to culture *Chaetoceros costatus* and *Chaetoceros socialis* in N-, P- and Si-deprived culture media and extract the collected biomass. Furthermore, the antioxidant properties and chemical profile of the extracts were compared to extracts from biomass cultured in a standard growth medium (F/2).

## 2. Results and Discussion

### 2.1. Total Phenolic Content & Antioxidant Potentials

**Figure 1:**
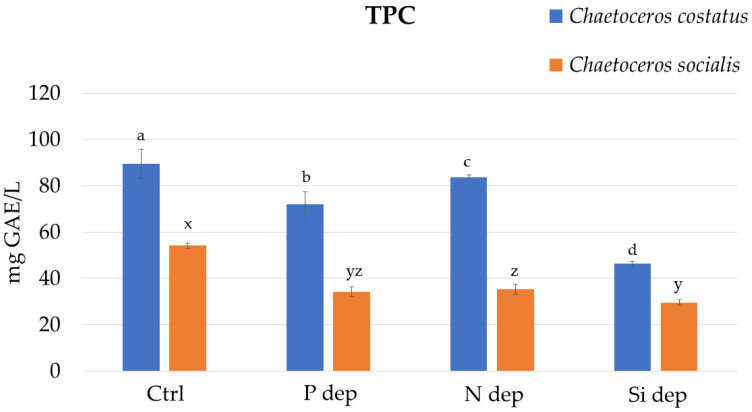
Total phenolic content (TPC) of the extracts from the biomass of *Chaetocerus costatus* and *Chaetocerus socialis* cultivated in standard F/2 medium (Ctrl) and under nutrient deprivation with phosphorus (P dep), nitrogen (N dep) and silica (Si dep). Letters a–d denote statistically significant differences (*p* < 0.05) between the extracts from *C. costatus* and letters x–z denote statistically significant differences (*p* < 0.05) between the extracts from *C. socialis*.

The TPC of *C. socialis* and *C. costatus* in a standard F/2 medium was 89.38 ± 6.21 and 54.17 ± 1.18 mg of gallic acid equivalent (GAE)/L, respectively. The lowest TPC results were found in the Si-deprived medium, an almost 2-fold decrease compared to the control medium (Figure 1).

In the extracts of *C. costatus*, TPC was in range from 46.25 ± 1.08 to 89.38 ± 6.21 mg GAE/L, being the highest for diatoms cultured in the control (F/2) medium. The TPC of *C. socialis* was significantly lower in all media, ranging from 29.58 ± 1.08 to 54.17 ± 1.18 mg GAE/L.

The phenolic compounds in diatom cells serve as a protective mechanism against oxidative stress that can be triggered by abiotic parameters such as a nutrient deficiency. It is known that the contents of phenolic compounds vary between different diatom species, as do the mechanisms of adaptation to nutrient stress [15]. In a study on the diatom *Phaeodactylum tricornutum* exposed to nitrogen deprivation (N−) for 15 days, significant differences in total polyphenol content were found between cultures compared to standard culture conditions (N+) [10]. *Phaeodactylum tricornutum* produced a higher phenolic content in N-enriched medium (3.07 ± 0.17 mg GAE/g DW) compared to *P. tricornutum* grown under N-limitation (1.12 ± 0.00 mg GAE/g DW). The same was observed in the species *C. costatus* and *C. socialis* from this study, where the phenolic content was significantly lower in all experimental groups exposed to nutrient deficiency (P, N and Si) compared to the control group. Previously, a positive correlation between antioxidant activity and TPC was found in microalgae *Tetraselmis marina* isolated from the northern Adriatic Sea [16]. Interestingly, in nutrient-deprived culture conditions, both TPC and pigments may contribute significantly to antioxidant capacity [17].

**Figure 2:**
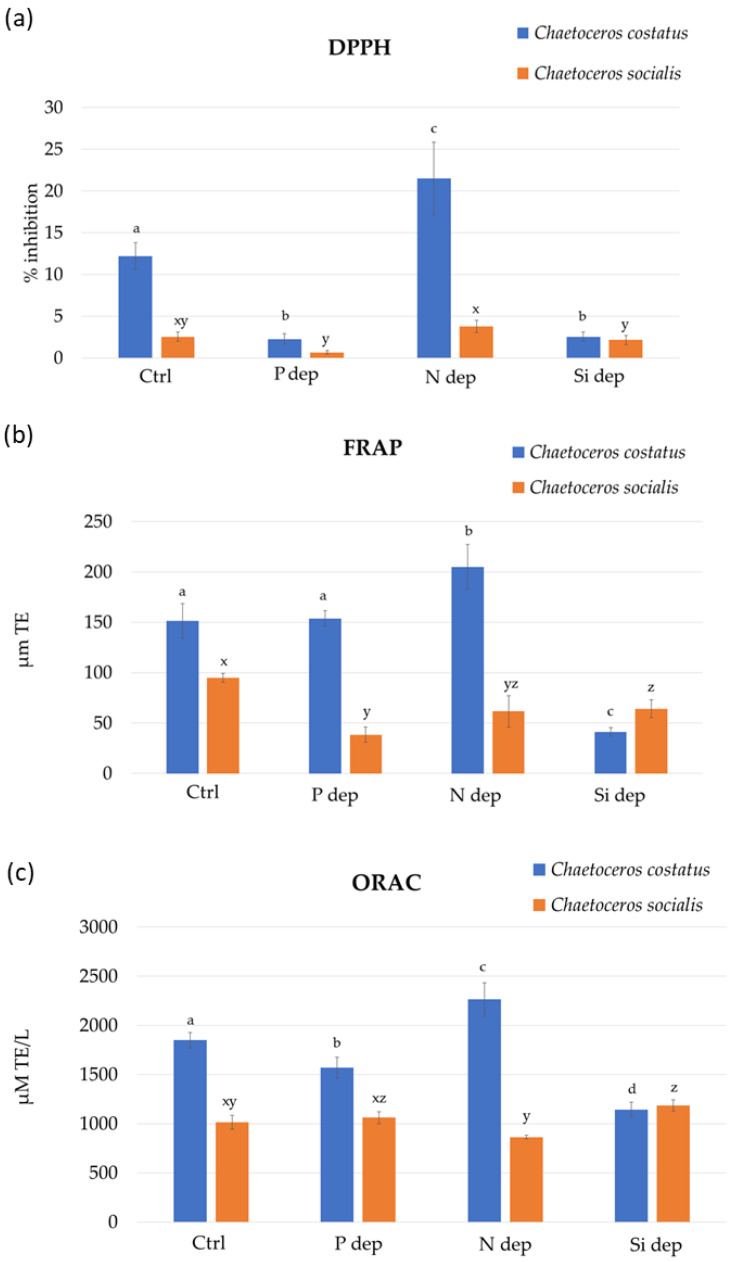
A 2,2-diphenyl-1-picrylhydrazyl radical scavenging ability (DPPH) (**a**); ferric reducing/antioxidant power (FRAP) (**b**); and oxygen radical absorbance capacity (ORAC) (**c**) for the extracts of *Chaetocerus costatus* and *Chaetocerus socialis* biomass cultured in standard F/2 medium (Ctrl) and under nutrient deprivation with phosphorus (P dep), nitrogen (N dep), and silica (Si dep). Letters a–d denote statistically significant differences (*p* < 0.05) between the extracts from *C. costatus* and letters x–z denote statistically significant differences (*p* < 0.05) between the extracts from *C. socialis*.

A comprehensive insight into the antioxidant potential of diatom extracts is shown in Figure 2. All three methods demonstrated the higher antioxidant potential of *C. costatus*. When cultured in N-deprived medium, the antioxidant potential of the *C. costatus* extracts were higher for all assays. However, this trend cannot be confirmed for *C. socialis*, as no clear correlation between the antioxidant activity and the culture medium can be established. The highest potential for the inhibition of the DPPH radical (21.52 ± 4.35% of inhibition) was found for *C. costatus* extracts cultured in the N-deprived medium, while the extract of diatoms cultured in the P-deprived medium showed the lowest results. For this antioxidant method, *C. socialis* extracts followed the same trend but with a lower inhibition. The FRAP results for the extracts of *C. costatus* ranged from 205.13 ± 22.21 to 41.03 ± 4.44 µM Trolox equivalents (TE), with the highest results obtained for the N-deprived medium, followed by the P-deprived medium and control medium, and the lowest in the Si-deprived medium. For the *C. socialis* extracts, the highest FRAP result (94.87 ± 4.44 µM TE) was observed in the control medium. The FRAP results decreased when nutrient deprivation was tested following the Si > N > P trend.

The results of ORAC obtained for *C. costatus* extracts ranged from 2264.63 ± 170.02 to 1143.78 ± 73.18 µM TE. Only extracts of the N-deprived medium showed higher results than the control medium. The growth of diatoms in P-deprived and Si-deprived media resulted in lower peroxyl radical inhibition of extracts for this species. However, the opposite results were observed for *C. socialis* extracts, where the N-deprived medium showed the lowest ORAC results (864.67 ± 18.16 µM TE), while P-deprived and Si-deprived media showed higher results than the control medium, 1062.63 ± 60.64 and 1185.08 ± 59.42 µM TE, respectively.

In all three tests, the highest antioxidant activity during N deprivation was observed in the species *C. costatus*, but the same trend was not recorded in the species *C. socialis*. Therefore, the relationship between nutrient deprivation and antioxidant properties should be observed at the strain level, as differences were found within microalgal groups, but also at the genus level. In a study by Curcuraci et al. [10], the antioxidant properties of the diatom *P. tricornutum* cultivated in nutrient deprivation media were estimated using DPPH and FRAP assays. Both assays revealed a statistically significantly lower antioxidant capacity for *P. tricornutum* from a medium with N deprivation. Furthermore, a lower ability to scavenge DPPH radicals and a decrease in reducing power were observed for the green microalgae *Dunaliela salina* from an N-deprived medium [18]. On the other hand, Jeyakumar et al. [19] observed the highest DPPH scavenging activity in the haptophyte *Isochrysis* sp. under N-deficient conditions with an inhibition of 85%, while the nitrogen-rich and control medium showed a lower inhibition of 75% and 64%, respectively.

### 2.2. Non-Target Screening of Non-Volatile Compounds in Ethanol Extract

The ethanolic extracts of the freeze-dried microalgae samples were analyzed by ultra-high-performance liquid chromatography-high-resolution mass spectrometry (UHPLC-ESI–HRMS). The compounds were identified based on the provided elemental composition in combination with MS/MS spectra with confidence levels 2 (probable structure) and 3 (possible structure) [20]. Out of the thirty-eight identified compounds, fourteen were pigments and derivatives, nineteen were fatty acid derivatives, and five were steroids and derivatives (Table 1).

The group of pigments and derivatives was the most diverse when comparing different growing conditions and microalgae species. Five chlorophyll *a* derivatives (pheophorbide *a* (no. 8), divinyl pheophytin *a* (no. 11), 15^1^-hydroxy-lactone-pheophytin *a* (no. 12), 13^2^-hydroxy-pheophytin *a* (no. 13) and pheophytin *a* (no. 14)); two chlorophyll *b* derivatives (pheophorbide *b* (no. 4) and pheophytin *b* (no. 10)); five xanthophylls and derivatives (no. 2–3, 5–7); one pheophytine derivative (no. 9) and monoterpene lactone (loliolide, no. 1) were detected (Table 1). Pheophytin *a* was the most abundant pigment derivative in all samples, except in the samples with Si-deprivation in both species. In the N-deprived samples of *C. costatus*, loliolide, pheophorbide *a*, pheophytin *b* and three chlorophyll *a* derivatives compounds no. 9, 12 and 13 were more abundant compared to the other samples. Also, in *C. costatus* extracts, a compound with a potential application in nutrition and pharmacy, fucoxanthin, was significantly higher in the P-deprived and N-deprived medium by 80% and 85%, respectively. The compound (3β)-3-HydroXystigmast-5-en-7-one, known for its antimalarial activity [21], was detected in *C. costatus* extracts cultured in the N-deprived medium. Similar results were detected for a pheophorbide in *C. socialis* extracts (higher values in P-deprived and N-deprived medium by 77% and 86%, respectively), a chlorophyll derivative known for its anti-cancer, antioxidant, immunostimulatory, neuroprotective and anti-inflammatory activity [22]. Collier and Grossman [23] found the degradation of chlorophyll as soon as nitrogen was removed from the medium when culturing cyanobacterium *Synechococcus* sp. It is very likely that these compounds contributed to the increased antioxidant activity in these samples, particularly in *C. costatus*. The monoterpene hydroxylactone loliolide, a photo-oxidative degradation product of carotenoids, such as fucoxanthin [24], is known as an antioxidant [25] and is widely distributed in macroalgae [26,27,28,29,30]. Menzel et al. [31] used it as a biomarker in haptophytes, diatoms, dinoflagellates, and eustigmatophytes while investigating the deposition of sapropel. Loliolide was detected in acetone and MeOH extracts of the Antarctic diatoms *Craspedostauros ineffabilis* and *C. zucchelli,* as well as in a supercritical CO_2_ extract of the green microalga *Tetradesmus obliquus* [32]. In the N-deprived sample, it was 1.2 times more abundant than in the control sample, 1.8 times more than in the P-deprived sample and 5.0 times more than in the Si-deprived sample. Loliolide has shown neuroprotective and anti-inflammatory activities [33] as well as anti-apoptosis and anti-scratching activities in human skin [29]. Pheophytines are the simplest derivatives of chlorophylls, in which the Mg atom is removed from the porphyrin ring. Further degradation leads to pheophorbides and their derivatives such as compounds no. 9 and 11–13. Both pheophytines and pheophorbides have shown antioxidant activities [22,34,35]. Pheophorbid *a* has shown anticancer [22], antiviral [36,37], anti-inflammatory [38], and antiparasitic activities [39].

In the group of fatty acid derivatives, there were eight primary fatty acid amides (PFAAs), with oleamide (no. 22) being the most abundant (Table 1). This PFAA has already been found as the dominant compound in green and brown macroalgae [28,40,41] and in the diatom *Skeletonema grevillei* [42]. Two other C:18 PFAAa were detected, linoleamide (no. 19) and stearamide (no. 23). In addition, two C:16 PFAAs, palmitoleamide (no. 18) and palmitamide (no. 20), one C:14 PFAA, myristamide (no. 16), C:20 PFAA, gondamide (no. 25), and C:22 PFAA, erucamide (no. 27) were detected. The abundance of all PFAAs was the highest in the control sample and decreased significantly in the samples deprived of nutrients, especially in the samples deprived of N in both species. PFAAs are bioactive signaling molecules that have the ability to bind to the receptors of many drugs. In this way, they could influence locomotion, angiogenesis, and sleep in mammals [43]. They have numerous bioactive properties, such as anticancer, antimicrobial, anthelmintic and antidiabetic activities [44,45,46,47]. Oleamide is the most studied PFAA and has promising potential against Alzheimer’s disease [43,44,45,48]. As secondary metabolites in algae, they probably play a role in defense against predators, as is the case in higher plants [44]. Four glycerophosphocholines (no. 28–31) were detected with the highest content in the control samples of both species. Three fatty acid esters (no. 17, 21, 24) and two diacylglycerols (no. 32–33) showed an increase in the N-deprived samples. A long-chain fatty acid C:20 (no. 26) and a C:16 sphingoid bases sphingolipid [49] hexadecasphinganine (no. 15), were also detected.

Five compounds (no. 33–38) were detected in the group of sterols and derivatives. Their contents varied from sample to sample and were not comparable between species. Studies have shown that microalgae can synthesize animal and plant sterols, with cholesterol, stigmasterol, ergosterol and champesterol being the most abundant [50]. In this study, champesterol (no. 35), deoxidised stigmasteol (stigmastatriene, no. 38) and three other derivatives (no. 34, 36, 37) were found. These compounds may increase antioxidant activity, as sterols are known to be antioxidant, anticarcinogenic, and anti-inflammatory compounds [51].

## 3. Materials and Methods

### 3.1. Experimental Design and Cultivation Conditions

The diatoms *C. costatus* (CIM935) and *C. socialis* (CIM929) were donated from the culture collection of the Center for Marine Research of the Ruđer Bošković Institute (Rovinj, Croatia). The strains were isolated from the northern Adriatic Sea.

The control groups (Ctrl) of both strains were cultivated in the standard F/2 medium, while the other three treatment groups were cultivated in phosphorus (P dep)-, nitrogen (N dep)- and silicon (Si dep)-deficient conditions (Table 2).

During the cultivation, all groups were held at a temperature of 18 °C, at a light intensity of 2500 lux (Led GNC Minu Deep AM140, Sicce, Pozzoleone, Italy) and a 16:8 light:dark cycle. The diatoms were cultured in cell culture flasks, each containing 500 mL of the nutrient medium and 20 mL of the diatom inoculum (10^5^ cells/mL). The cultivation of each treatment group was carried out in two replicates.

### 3.2. Harvesting and Extraction

The diatom biomass was harvested at the stationary growth phase by the filtration trough glass microfiber filters (Grade GF/F Whatman) at a pressure of 3.21 psi. The collected biomass was transferred to falcon tubes with cell scrapers and freeze-dried (FreeZone 2.5, Labconco, Kansas City, MO, USA) [53].

Ultrasound-assisted extraction (UAE) of the freeze-dried biomass was performed in an ultrasonic bath (DU-100 Digital ultrasonic cleaner, Giorgio Bormac, Carpi, Italy) with 70% ethanol at a frequency of 40 kHz and 50 °C for 1 h. The samples were centrifuged (Rotafix 32A, Hettich, Tuttlingen, Germany) for 5 min at room temperature and 3220 x *g* (4000 rpm) and the obtained supernatants were filtered through a 0.45 µM mixed cellulose ester filter (LGG, Meckenheim, Germany) and dried by centrifugal evaporator (RC10-22, Jouan, Herblain, France).

### 3.3. Antioxidation Assays

The dried extracts of both diatom strains were dissolved in 70% ethanol at a concentration of 20 mg/mL prior to the analyses.

The Folin–Ciocalteu method was used to determine the total phenolic content (TPC) of the diatom extracts [54]. In summary, 25 µL of the *Chaetocerus* extracts and 1.5 mL of distilled water were combined with 25 µL of the Folin–Ciocalteu reagent. The reagent was added, and the mixtures were agitated and left for a one minute before adding 375 µL of 20% sodium carbonate solution and 475 µL of distilled water. Samples were kept in the dark at room temperature for two hours, and measurements were performed with a spectrophotometer (UV-1900i, Shimadzu, Tokyo, Japan) at an absorbance of 765 nm. The results were expressed in milligrams or gallic acid equivalents (GAE) per L of extract.

The ability to scavenge 2,2-diphenyl-1-picrylhydrazyl (DPPH) radicals, ferric reducing/antioxidant power (FRAP), and the oxygen radical absorbance capacity (ORAC) were used to evaluate the antioxidant potential of *C. costatus* and *C. socialis* extracts.

The reducing activity was measured with FRAP [55]. The absorbance of 300 µL of FRAP reagent solution was measured at 592 nm using a plate reader (SynergyHTX Multi-Mode Reader, BioTek Instruments, Inc., Winooski, VT, USA) in 96-well microplates. The change of absorbance was measured 4 min after adding 10 µL of the sample to the FRAP reagent. The absorbance of the FRAP reagent before the addition of the sample and four minutes afterward was compared with a value determined for the Trolox reference solution and expressed in µM TE.

The ability of the diatom extracts to scavenge DPPH radicals was also assessed [53]. Measurements were performed at 517 nm after adding of 290 µL of DPPH radical solution with an initial absorbance of 1.2 nm in the microplate wells. A plate reader was used to measure the decreased in absorbance one hour after adding 10 µL of the *Chaetocerus* extracts to the wells. The percentage of DPPH radical inhibition that the diatom extracts were able to inhibit (% inhibition) was used to quantify their antioxidant activity.

The ORAC assay was performed according to previously described protocols [56,57]. A volume of 25 µL of the diatom extracts was added to the wells of a microtiter plate containing 150 µL of 4.2 mM fluorescein (3′,6′-dihydroxyspiro[isobenzofuran-1(3H),9′-[9H] xanthan]-3-one). After thermostating at 37 °C for 30 min, 25 µL of AAPH (2,2′-azobis (2-amidinopropane) dihydrochloride) was added to the plates. Excitation and emission wavelength measurements were performed at 485 and 520 nm every minute for eighty minutes and the results were expressed in µM Trolox equivalents (µM TE). All mentioned assays were performed in triplicate.

### 3.4. Ultra-High-Performance Liquid Chromatography-High-Resolution Mass Spectrometry (UHPLC-ESI-HRMS) of Ethanol Extract

ExionLC AD UHPLC system (AB Sciex, Concord, ON, Canada) connected to a quadrupole time-of-flight (Q-TOF) mass spectrometer TripleTOF 6600+ (AB Sciex, Concord, ON, Canada) with a duospray ion source was used for the UHPLC-ESI-HRMS analyses. The Acquity UPLC BEH Phenyl-Hexyl analytical column (Waters, Milford, MA, USA) 2.1 mm × 100 mm with a particle size of 1.7 µM was used for the chromatographic separation of the compounds. Water, as a mobile phase A, and acetonitrile, as a mobile phase B, both contained 0.1% formic acid. The flow rate of 0.4 mL/min and the oven temperature of 30 °C were constant throughout the analysis. Elution started at 2% B and was held for 0.6 min, followed by a linear B gradient to 100% until 18.5 min. From 18.5–25 min, elution was again isocratic at 100% B. Electrospray ionization was set in positive mode (ESI+) with collision-induced dissociation (CID) in information-dependent acquisition mode (IDA) for MS/MS mass spectra acquisition. A detailed description of the parameters can be found in our previous article [40]. The mass spectrometer data were processed using ACD/Spectrus Processor 2021.1.0 software (ACD/Labs, Toronto, ON, Canada). Based on the mass spectra and the reported elemental compositions of the compounds combined with the results of the search in the MassBank, Lipid Maps, ChemSpider and ChEBI databases, the identification of the compounds was proposed.

### 3.5. Statistical Analysis

Analyses of variance (one-way ANOVA followed by Fisher’s least significant difference test) were used to express the statistical difference for the results of the TPC, FRAP, DPPH and ORAC assays between the results obtained for different culture media of each species [58]. The analyses were performed with Statgraphics Centurion-Ver. 16.1.11 (StatPoint Technologies, Inc., Warrenton, VA, USA).

## 4. Conclusions

Nutrient deprivation with phosphorus, nitrogen, and silicon in the species *C. costatus* and *C. socialis* resulted in a lower TPC. On the other hand, a significant increase in antioxidant capacity was observed during nitrogen deprivation in *C. costatus*, while the same trend was not observed in *C. socialis*. Among the identified compounds, pigments and derivatives, fatty acid derivatives as well as sterols and derivatives were detected in both species. Higher occurrences of pigment derivatives, loliolide, pheophorbide *a*, pheophytin *b*, and three chlorophyll *a* derivatives, which are possibly responsible for antioxidant activity, were observed in the N-deprived medium. Among the dominant compounds from the group of fatty acids, oleamide was detected and, in contrast to the pigments, deprivation with N led to a significant decrease in the content of PFAAs in both species. For this reason, the influence of single-nutrient deprivation on the chemical composition and antioxidant activity, even at the level of a single genus, is inconclusive. Certainly, future research should investigate cell growth rate and biomass yield in a nutrient-deprived medium and their correlation to specific compound contents, and whether the combination of nutrient deprivation with another stress trigger can further increase the synthesis of these bioactive compounds to further explore their overall bioactive potential.

## Figures and Tables

**Table 1:** Major non-volatile compounds in *Chaetoceros costatus* and *Chaetoceros socialis* ethanol extracts identified using high-performance liquid chromatography–high-resolution mass spectrometry with electrospray ionisation (UHPLC-ESI–HRMS). Ctrl—control sample; samples of biomass cultured under nutrient deprivation with phosphorus (P dep), nitrogen (N dep), and silica (Si dep).

No.	Compound Name	Mass	[M+H]+	Molecular Formula	t_R_ (min)	Mass Difference (ppm)	Peak Area (Arbitrary Units)							
							*Chaetoceros costatus*				*Chaetoceros socialis*			
							Ctrl	P dep	N dep	Si dep	Ctrl	P dep	N dep	Si dep
Pigments and Derivatives														
1	Loliolide	196.110	197.11722	C_11_H_16_O_3_	5.866	0.1	1.65 × 10^6^	1.06 × 10^6^	1.95 × 10^6^	3.87 × 10^5^	2.53 × 10^5^	1.32 × 10^5^	1.62 × 10^5^	1.54 × 10^5^
2	Apo-10-fucoxanthinal	424.261	425.26864	C_27_H_36_O_4_	9.514	1	1.50 × 10^5^	4.13 × 10^4^	7.99 × 10^4^	-	6.09 × 10^4^	6.98 × 10^3^	5.54 × 10^4^	-
3	Halocynthiaxanthin acetate	640.413	641.42005	C_42_H_56_O_5_	12.364	2	6.39 × 10^5^	1.88 × 10^5^	3.89 × 10^5^	-	3.64 × 10^5^	2.26 × 10^4^	4.21 × 10^5^	2.41 × 10^4^
4	Pheophorbide *b*	606.248	607.25511	C3_5_H_34_N_4_O_6_	12.371	1	6.33 × 10^4^	4.83 × 10^4^	5.82 × 10^4^	7.13 × 10^3^	1.43 × 10^4^	6.17 × 10^3^	9.87 × 10^3^	4.85 × 10^2^
5	Fucoxanthin	658.423	659.43062	C_42_H_58_O_6_	12.385	2.1	1.18 × 10^5^	9.73 × 10^4^	7.69 × 10^4^	-	6.90 × 10^5^	4.34 × 10^4^	7.97 × 10^5^	6.02 × 10^3^
6	Diatoxanthin	566.412	567.41966	C_4_0H_54_O_2_	12.772	0.1	4.21 × 10^4^	3.90 × 10^4^	1.22 × 10^4^	1.86 × 10^4^	6.43 × 10^4^	3.52 × 10^4^	3.69 × 10^4^	1.92 × 10^4^
7	Fucoxanthinol	616.413	617.42005	C_4_0H_56_O_5_	12.836	2.8	9.89 × 10^4^	5.03 × 10^4^	6.45 × 10^4^	4.14 × 10^4^	5.75 × 10^3^	9.44 × 10^3^	6.02 × 10^3^	9.99 × 10^3^
8	Pheophorbide *a*	592.269	593.27585	C_35_H_36_N_4_O_5_	13.125	1.3	5.18 × 10^6^	1.26 × 10^6^	5.75 × 10^6^	2.78 × 10^4^	2.50 × 10^5^	3.91 × 10^4^	1.24 × 10^5^	4.15 × 10^3^
9	3-[21-Methoxycarbonyl-4,8,13,18-tetramethyl-20-oxo-9,14-divinyl-3,4-didehydro-3-24,25-dihydrophorbinyl]propanoic acid	588.237	589.24455	C_35_H_32_N_4_O_5_	13.247	3	9.06 × 10^5^	3.67 × 10^5^	9.17 × 10^5^	5.31 × 10^4^	1.51 × 10^5^	9.19 × 10^4^	1.66 × 10^5^	1.32 × 10^4^
10	Pheophytin *b*	884.545	885.55246	C_55_H_72_N_4_O_6_	18.463	3.5	1.50 × 10^5^	2.75 × 10^4^	1.62 × 10^5^	-	9.22 × 10^4^	5.06 × 10^3^	3.12 × 10^5^	-
11	Divinyl pheophytin *a*	868.550	869.55755	C_55_H_72_N_4_O_5_	18.721	2.4	4.20 × 10^5^	1.27 × 10^5^	2.69 × 10^5^	-	1.04 × 10^5^	9.56 × 10^3^	2.03 × 10^5^	-
12	15^1^-hydroxy-lactone-pheophytin *a*	902.556	903.56303	C_55_H_74_N_4_O_7_	18.842	0.8	9.45 × 10^5^	5.63 × 10^5^	1.20 × 10^6^	2.67 × 10^3^	4.66 × 10^5^	5.32 × 10^4^	4.43 × 10^5^	2.13 × 10^3^
13	13^2^-hydroxy-pheophytin *a*	886.561	887.56811	C_55_H_74_N_4_O_6_	18.859	0.1	4.03 × 10^5^	5.46 × 10^5^	5.44 × 10^5^	1.64 × 10^4^	4.65 × 10^5^	5.03 × 10^4^	4.14 × 10^5^	1.99 × 10^4^
14	Pheophytin *a*	870.566	871.5732	C_55_H_74_N_4_O_5_	19.071	0.4	2.40 × 10^7^	4.74 × 10^6^	1.91 × 10^7^	5.27 × 10^4^	3.31 × 10^6^	2.12 × 10^5^	3.98 × 10^6^	5.24 × 10^4^
Fatty Acid Derivatives														
15	Hexadecasphinganine	273.267	274.27406	C_16_H_35_NO_2_	6.24	0.6	8.20 × 10^6^	7.33 × 10^6^	5.69 × 10^6^	5.19 × 10^6^	9.40 × 10^6^	5.76 × 10^6^	6.39 × 10^6^	4.33 × 10^6^
16	Myristamide (Tetradecanamide)	227.225	228.23219	C_14_H_29_NO	10.328	0.6	2.59 × 10^6^	1.76 × 10^6^	1.41 × 10^6^	2.15 × 10^6^	3.69 × 10^6^	1.98 × 10^6^	2.89 × 10^6^	2.04 × 10^6^
17	Monomyristin (2,3-Dihydroxypropyl tetradecanoate)	302.246	303.25299	C_17_H_34_O_4_	10.641	6.4	7.80 × 10^4^	8.87 × 10^4^	1.86 × 10^5^	8.32 × 10^4^	6.21 × 10^4^	2.08 × 10^5^	2.09 × 10^5^	1.53 × 10^5^
18	Palmitoleamide (Hexadec-9-enamide)	253.241	254.24784	C_16_H_31_NO	10.743	0.2	6.10 × 10^6^	4.33 × 10^6^	3.30 × 10^6^	4.44 × 10^6^	9.16 × 10^6^	4.91 × 10^6^	6.28 × 10^6^	4.43 × 10^6^
19	Linoleamide (Octadeca-9,12-dienamide)	279.256	280.26349	C_18_H_33_NO	11.182	0.1	5.91 × 10^6^	4.60 × 10^6^	1.31 × 10^6^	5.05 × 10^6^	1.14 × 10^7^	6.11 × 10^6^	8.14 × 10^6^	4.86 × 10^6^
20	Palmitamide (Hexadecanamide)	255.256	256.26349	C_16_H_33_NO	11.437	0.2	1.48 × 10^7^	1.22 × 10^7^	9.33 × 10^6^	1.11 × 10^7^	2.29 × 10^7^	1.30 × 10^7^	1.54 × 10^7^	1.17 × 10^7^
21	Monopalmitin (2,3-Dihydroxypropyl hexadecanoate)	330.277	331.28429	C_19_H_38_O_4_	11.71	0.1	3.62 × 10^6^	3.49 × 10^6^	3.98 × 10^6^	2.64 × 10^6^	4.36 × 10^6^	3.88 × 10^6^	4.49 × 10^6^	2.70 × 10^6^
22	Oleamide (Octadec-9-enamide)	281.272	282.27914	C_18_H_35_NO	11.813	0.3	1.06 × 10^8^	8.81 × 10^7^	6.67 × 10^7^	7.65 × 10^7^	9.55 × 10^7^	5.47 × 10^7^	1.03 × 10^7^	7.53 × 10^7^
23	Stearamide (Octadecanamide)	283.288	284.29479	C_18_H_37_NO	12.506	0.8	7.89 × 10^6^	6.91 × 10^6^	5.23 × 10^6^	6.17 × 10^6^	1.27 × 10^7^	6.93 × 10^6^	9.03 × 10^6^	7.06 × 10^6^
24	Monostearin (2,3-Dihydroxypropyl octadecanoate)	358.308	359.31559	C_21_H_42_O_4_	12.765	0.6	3.70 × 10^6^	3.51 × 10^6^	3.98 × 10^6^	2.27 × 10^6^	4.16 × 10^6^	2.92 × 10^6^	4.46 × 10^6^	2.46 × 10^6^
25	Gondamide (Icos-11-enamide)	309.303	310.31044	C_2_0H_39_NO	12.796	1.7	1.29 × 10^6^	1.01 × 10^6^	7.99 × 10^5^	1.03 × 10^6^	2.29 × 10^6^	1.07 × 10^6^	1.61 × 10^6^	1.09 × 10^6^
26	Arachidonic acid (Icosa-5,8,11,14-tetraenoic acid)	304.240	305.24751	C_2_0H_32_O_2_	13.16	0.3	3.85 × 10^4^	5.46 × 10^4^	5.14 × 10^4^	5.25 × 10^4^	5.75 × 10^4^	5.61 × 10^4^	5.50 × 10^4^	3.53 × 10^4^
27	Erucamide (Docos-13-enamide)	337.334	338.34174	C_22_H_43_NO	13.787	0.6	2.00 × 10^6^	1.47 × 10^6^	1.30 × 10^6^	9.99 × 10^5^	2.09 × 10^6^	1.16 × 10^6^	1.80 × 10^6^	1.01 × 10^6^
28	1-(9-Octadecenoyl)-2-(9-pentadecenoyl)-glycero-3-phosphocholine	743.547	744.55378	C_41_H_78_NO_8_P	15.863	2.8	7.52 × 10^4^	1.74 × 10^4^	4.53 × 10^4^	3.51 × 10^4^	8.82 × 10^4^	6.16 × 10^4^	8.76 × 10^4^	5.54 × 10^4^
29	1-(11,14-Eicosadienoyl)-2-heptadecanoyl-glycero-3-phosphoserine	801.552	802.55926	C_43_H_8_0NO_1_0P	16.300	0.1	1.95 × 10^5^	1.22 × 10^5^	1.45 × 10^5^	7.31 × 10^4^	1.68 × 10^5^	1.07 × 10^5^	1.63 × 10^5^	6.01 × 10^4^
30	1-Octadecanoyl-2-(9,12-heptadecadienoyl)-glycero-3-phosphocholine	771.578	772.58508	C_43_H_82_NO_8_P	16.403	2.6	7.63 × 10^4^	1.31 × 10^4^	3.56 × 10^4^	5.58 × 10^4^	9.23 × 10^4^	8.82 × 10^4^	8.22 × 10^4^	7.74 × 10^4^
31	1-(9-Octadecenoyl)-2-(9-nonadecenoyl)-glycero-3-phosphocholine	799.609	800.61638	C_45_H_86_NO_8_P	16.737	1.5	7.04 × 10^4^	3.50 × 10^4^	3.33 × 10^4^	2.29 × 10^4^	6.11 × 10^4^	4.75 × 10^4^	5.33 × 10^4^	1.31 × 10^4^
32	Dipalmitin	568.507	569.51395	C_35_H_68_O_5_	17.729	1.2	3.60 × 10^4^	3.09 × 10^4^	3.70 × 10^4^	1.52 × 10^4^	2.53 × 10^4^	2.70 × 10^4^	3.39 × 10^4^	3.20 × 10^4^
33	1-Octadecanoyl-2-hexadecanoyl-*sn*-glycerol	596.538	597.54525	C_37_H_72_O_5_	18.276	1.6	2.30 × 10^4^	1.76 × 10^4^	2.54 × 10^4^	8.29 × 10^3^	1.96 × 10^4^	1.59 × 10^4^	2.09 × 10^4^	1.75 × 10^4^
Steroids and Derivatives														
34	Chola-5,22-dien-3-ol	342.292	343.29954	C_24_H_38_O	7.426	4.8	1.30 × 10^5^	1.14 × 10^5^	1.36 × 10^5^	1.37 × 10^5^	1.01 × 10^5^	8.97 × 10^4^	1.54 × 10^5^	1.60 × 10^5^
35	Campesterol	400.371	401.37779	C_28_H_48_O	7.944	0.8	9.75 × 10^3^	4.13 × 10^2^	7.92 × 10^3^	5.52 × 10^3^	1.20 × 10^4^	4.19 × 10^3^	8.97 × 10^3^	4.80 × 10^3^
36	24-Hydroperoxy-24-vinyl-cholesterol	444.360	445.36762	C_29_H_48_O_3_	14.914	0.2	3.95 × 10^4^	2.26 × 10^4^	4.35 × 10^4^	2.08 × 10^4^	7.14 × 10^3^	1.45 × 10^4^	9.19 × 10^3^	1.14 × 10^4^
37	(3β)-3-Hydroxystigmast-5-en-7-one	428.365	429.37271	C_29_H_48_O_2_	15.434	3.8	1.70 × 10^5^	1.01 × 10^5^	1.79 × 10^5^	6.52 × 10^4^	3.07 × 10^4^	-	3.02 × 10^4^	3.43 × 10^4^
38	Stigmastatriene	394.360	395.36723	C_29_H_46_	15.609	1.9	2.33 × 10^4^	3.16 × 10^4^	1.99 × 10^4^	2.04 × 10^4^	1.02 × 10^4^	4.29 × 10^4^	2.01 × 10^4^	2.33 × 10^4^

**Table 2:** The composition of a nutrient medium used for the cultivation of the strains *Chaetocerus costatus* and *Chaetocerus socialis* under nutrient deprivation with phosphorus (P dep), nitrogen (N dep), and silica (Si dep).

Treatment Group	Composition
Control group (Ctrl)	The F/2 medium was prepared according to the previously described recipe [52]
P dep	Based on F/2 medium without addition of NaH_2_PO_4_·H_2_O
N dep	Based on F/2 medium without addition of NaNO_3_
Si dep	Based on F/2 medium without addition of Na_2_SiO_3_·9H_2_O

## Data Availability

The data presented in this study are available on request from the corresponding author.

## References

[1] Rizwan M., Mujtaba G., Memon S.A., Lee K., Rashid N. (2018). Exploring the potential of microalgae for new biotechnology applications and beyond: A review. Renew. Sustain. Energy Rev..

[2] Field C.B., Behrenfeld M.J., Randerson J.T., Falkowski P. (1998). Primary production of the biosphere: Integrating terrestrial and oceanic components. Science.

[3] Lin Q., Zhuo W.H., Wang X.W., Chen C.P., Gao Y.H., Liang J.R. (2018). Effects of fundamental nutrient stresses on the lipid accumulation profiles in two diatom Species *Thalassiosira weissflogii* and *Chaetoceros muelleri*. Bioprocess Biosyst. Eng..

[4] Gatamaneni B.L., Orsat V., Lefsrud M. (2018). Factors affecting growth of various microalgal species. Environ. Eng. Sci..

[5] Vello V., Phang S.-M., Poong S.-W., Lim Y.-K., Ng F.-L., Shanmugam J., Gopal M. (2023). New Report of *Halamphora subtropica* (Bacillariophyta) from the Strait of Malacca and its growth and biochemical characterisation under nutrient deprivation. Reg. Stud. Mar. Sci..

[6] Martin-Jézéquel V., Hildebrand M., Brzezinski M.A. (2000). Silicon metabolism in diatoms: Implications for growth. J. Phycol..

[7] Orefice I., Musella M., Smerilli A., Sansone C., Chandrasekaran R., Corato F., Brunet C. (2019). Role of nutrient concentrations and water movement on diatom’s productivity in culture. Sci. Rep..

[8] Shrestha R.P., Hildebrand M. (2015). Evidence for a regulatory role of diatom silicon transporters in cellular silicon responses. Eukaryot. Cell.

[9] Lovio-Fragoso J.P., de Jesús-Campos D., López-Elías J.A., Medina-Juárez L.Á., Fimbres-Olivarría D., Hayano-Kanashiro C. (2021). Biochemical and molecular aspects of phosphorus limitation in diatoms and their relationship with biomolecule accumulation. Biology.

[10] Curcuraci E., Manuguerra S., Messina C.M., Arena R., Renda G., Ioannou T., Amato V., Hellio C., Barba F.J., Santulli A. (2022). Culture conditions affect antioxidant production, metabolism and related biomarkers of the microalgae *Phaeodactylum tricornutum*. Antioxidants.

[11] Smith S.R., Glé C., Abbriano R.M., Traller J.C., Davis A., Trentacoste E., Vernet M., Allen A.E., Hildebrand M. (2016). Transcript level coordination of carbon pathways during silicon starvation-induced lipid accumulation in the diatom *Thalassiosira pseudonana*. N. Phytol..

[12] Yu S.J., Shen X.F., Ge H.Q., Zheng H., Chu F.F., Hu H., Zeng R.J. (2016). Role of sufficient phosphorus in biodiesel production from diatom *Phaeodactylum tricornutum*. Appl. Microbiol. Biotechnol..

[13] Gao Y., Yang M., Wang C. (2013). Nutrient deprivation enhances lipid content in marine microalgae. Bioresour. Technol..

[14] Lovio Fragoso J., Hayano Kanashiro C., Lopez Elias J. (2019). Effect of different phosphorus concentrations on growth and biochemical composition of *Chaetoceros muelleri*. Lat. Am. J. Aquat. Res..

[15] Goiris K., Van Colen W., Wilches I., León-Tamariz F., De Cooman L., Muylaert K. (2015). Impact of nutrient stress on antioxidant production in three species of microalgae. Algal Res..

[16] Trentin R., Custódio L., Rodrigues M.J., Moschin E., Sciuto K., da Silva J.P., Moro I. (2022). Total phenolic levels, In vitro antioxidant properties, and fatty acid profile of two microalgae, *Tetraselmis marina* strain IMA043 and *Naviculoid* diatom strain IMA053, isolated from the North Adriatic Sea. Mar. Drugs.

[17] Kuczynska P., Jemiola-Rzeminska M., Strzalka K. (2015). Photosynthetic pigments in diatoms. Mar. Drugs.

[18] Singh P., Baranwal M., Reddy S.M. (2016). Antioxidant and cytotoxic activity of carotenes produced by *Dunaliella salina* under stress. Pharm. Biol..

[19] Jeyakumar B., Asha D., Varalakshmi P., Kathiresan S. (2020). Nitrogen repletion favors cellular metabolism and improves eicosapentaenoic acid production in the marine microalga *Isochrysis* Sp. CASA CC 101. Algal Res..

[20] Blaženović I., Kind T., Ji J., Fiehn O. (2018). software tools and approaches for compound identification of LC-MS/MS data in metabolomics. Metabolites.

[21] Indriani I., Aminah N.S., Puspaningsih N.N.T. (2020). Antiplasmodial Activity of stigmastane steroids from *Dryobalanops oblongifolia* stem bark. Open Chem..

[22] Saide A., Lauritano C., Ianora A. (2020). Pheophorbide a: State of the art. Mar. Drugs.

[23] Collier J.L., Grossman A.R. (1992). Chlorosis induced by nutrient deprivation in *Synechococcus* Sp. strain PCC 7942: Not all bleaching is the same. J. Bacteriol..

[24] Repeta D.J. (1989). Carotenoid diagenesis in recent marine sediments: II. degradation of fucoxanthin to loliolide. Geochim. Cosmochim. Acta.

[25] Percot A., Yalçin A., Aysel V., Erduǧan H., Dural B., Güven K.C. (2009). Loliolide in marine algae. Nat. Prod. Res..

[26] El Hattab M., Culioli G., Valls R., Richou M., Piovetti L. (2008). Apo-fucoxanthinoids and loliolide from the brown alga *Cladostephus spongiosus* f. *verticillatus* (Heterokonta, Sphacelariales). Biochem. Syst. Ecol..

[27] Radman S., Cikoš A.M., Flanjak I., Babić S., Čižmek L., Šubarić D., Čož-Rakovac R., Jokić S., Jerković I. (2021). Less polar compounds and targeted antioxidant potential (in vitro and in vivo) of *Codium adhaerens* c. Agardh 1822. Pharmaceuticals.

[28] Radman S., Čižmek L., Babić S., Cikoš A.M., Čož-Rakovac R., Jokić S., Jerković I. (2022). Bioprospecting of less-polar fractions of *Ericaria crinita* and *Ericaria amentacea*: Developmental toxicity and antioxidant activity. Mar. Drugs.

[29] Park S.H., Kim D.S., Kim S., Lorz L.R., Choi E., Lim H.Y., Hossain M.A., Jang S.G., Choi Y.I., Park K.J. (2019). Loliolide presents antiapoptosis and antiscratching effects in human keratinocytes. Int. J. Mol. Sci..

[30] Yang X., Kang M.-C., Lee K.-W., Kang S.-M., Lee W.-W., Jeon Y.-J. (2011). Antioxidant activity and cell protective effect of loliolide isolated from *Sargassum ringgoldianum* Subsp. *coreanum*. Algae.

[31] Menzel D., Van Bergen P.F., Schouten S., Sinninghe Damsté J.S. (2003). Reconstruction of changes in export productivity during pliocene sapropel deposition: A biomarker approach. Palaeogeogr. Palaeoclimatol. Palaeoecol..

[32] Vladić J., Jerković I., Radman S., Jazić J.M., Ferreira A., Maletić S., Gouveia L. (2022). Supercritical CO_2_ extract from microalga *Tetradesmus obliquus*: The effect of high-pressure pre-treatment. Molecules.

[33] Silva J., Alves C., Martins A., Susano P., Simões M., Guedes M., Rehfeldt S., Pinteus S., Gaspar H., Rodrigues A. (2021). Loliolide, a new therapeutic option for neurological diseases? In vitro neuroprotective and anti-inflammatory activities of a monoterpenoid lactone isolated from *Codium tomentosum*. Int. J. Mol. Sci..

[34] Lanfer-Marquez U.M., Barros R.M.C., Sinnecker P. (2005). Antioxidant activity of chlorophylls and their derivatives. Food Res. Int..

[35] Hsu C.-Y., Chao P.-Y., Hu S.-P., Yang C.-M. (2013). The antioxidant and free radical scavenging activities of chlorophylls and pheophytins. Food Nutr. Sci..

[36] Ohta S., Ono F., Shiomi Y., Nakao T., Aozasa O., Nagate T., Kitamura K., Yamaguchi S., Nishi M., Miyata H. (1998). Anti-*Herpes simplex* virus substances produced by the marine green alga, *Dunaliella primolecta*. J. Appl. Phycol..

[37] Ratnoglik S.L., Aoki C., Sudarmono P., Komoto M., Deng L., Shoji I., Fuchino H., Kawahara N., Hotta H. (2014). Antiviral activity of extracts from *Morinda citrifolia* leaves and chlorophyll catabolites, pheophorbide *a* and pyropheophorbide *a*, against Hepatitis C virus. Microbiol. Immunol..

[38] Lauritano C., Helland K., Riccio G., Andersen J.H., Ianora A., Hansen E.H. (2020). Lysophosphatidylcholines and chlorophyll-derived molecules from the diatom *Cylindrotheca closterium* with anti-inflammatory activity. Mar. Drugs.

[39] Miranda N., Volpato H., da Silva Rodrigues J.H., Caetano W., Ueda-Nakamura T., de Oliveira Silva S., Nakamura C.V. (2017). The photodynamic action of pheophorbide a induces cell death through oxidative stress in *Leishmania amazonensis*. J. Photochem. Photobiol. B Biol..

[40] Radman S., Cikoš A.M., Babić S., Čižmek L., Čož-Rakovac R., Jokić S., Jerković I. (2022). In vivo and in vitro antioxidant activity of less polar fractions of *Dasycladus vermicularis* (Scopoli) Krasser 1898 and the chemical composition of fractions and macroalga volatilome. Pharmaceuticals.

[41] Radman S., Čagalj M., Šimat V., Jerković I. (2023). Seasonal monitoring of volatiles and antioxidant activity of brown alga *Cladostephus spongiosus*. Mar. Drugs.

[42] Frleta R., Popović M., Smital T., Šimat V. (2022). Comparison of growth and chemical profile of diatom *Skeletonema grevillei* in bioreactor and incubation-shaking cabinet in two growth phases. Mar. Drugs.

[43] Farrell E.K., Chen Y., Barazanji M., Jeffries K.A., Cameroamortegui F., Merkler D.J. (2012). Primary fatty acid amide metabolism: Conversion of fatty acids and an ethanolamine in N 18TG 2 and SCP Cells. J. Lipid Res..

[44] Tanvir R., Javeed A., Rehman Y. (2018). Fatty acids and their amide derivatives from endophytes: New therapeutic possibilities from a hidden source. FEMS Microbiol. Lett..

[45] D’Oca C.D.R.M., Coelho T., Marinho T.G., Hack C.R.L., Da Costa Duarte R., Da Silva P.A., D’Oca M.G.M. (2010). Synthesis and antituberculosis activity of new fatty acid amides. Bioorganic Med. Chem. Lett..

[46] Kabara J.J., Swieczkowski D.M., Conley A.J., Truant J.P. (1972). Fatty acids and derivatives as antimicrobial agents. Antimicrob. Agents Chemother..

[47] Dembitsky V.M. (2022). Microbiological aspects of unique, rare, and unusual fatty acids derived from natural amides and their pharmacological profile. Microbiol. Res..

[48] Ano Y., Ozawa M., Kutsukake T., Sugiyama S., Uchida K., Yoshida A., Nakayama H. (2015). Preventive effects of a fermented dairy product against Alzheimer’s disease and identification of a novel oleamide with enhanced microglial phagocytosis and anti-inflammatory activity. PLoS ONE.

[49] Fahy E., Subramaniam S., Brown H.A., Glass C.K., Merrill A.H., Murphy R.C., Raetz C.R.H., Russell D.W., Seyama Y., Shaw W. (2005). A comprehensive classification system for lipids. J. Lipid Res..

[50] Fagundes M.B., Vendruscolo R.G., Wagner R. (2020). Sterols from Microalgae.

[51] Volkman J.K., Borowitzka M.A., Beardall J., Raven J.A. (2016). The Physiology of Microalgae.

[52] Andersen R.A. (2005). Algal Culturing Techniques.

[53] Frleta Matas R., Popović M., Čagalj M., Šimat V. (2023). The marine diatom *Thalassiosira rotula*: Chemical profile and antioxidant activity of hydroalcoholic extracts. Front. Mar. Sci..

[54] Amerine M.A., Ough C.S. (1980). Methods Analysis of Musts and Wines.

[55] Benzie I.F.F., Strain J.J. (1996). The ferric reducing ability of plasma (FRAP) as a measure of “antioxidant power”: The FRAP assay. Anal. Biochem..

[56] Prior R.L., Hoang H., Gu L., Wu X., Bacchiocca M., Howard L., Hampsch-Woodill M., Huang D., Ou B., Jacob R. (2003). Assays for hydrophilic and lipophilic antioxidant capacity (oxygen radical absorbance capacity (ORAC_FL_)) of plasma and other biological and food samples. J. Agric. Food Chem..

[57] Burčul F., Generalić Mekinić I., Radan M., Rollin P., Blažević I. (2018). Isothiocyanates: Cholinesterase inhibiting, antioxidant, and anti-inflammatory activity. J. Enzyme Inhib. Med. Chem..

[58] Šimat V., Vlahović J., Soldo B., Generalić Mekinić I., Čagalj M., Hamed I., Skroza D. (2020). Production and characterization of crude oils from seafood processing by-products. Food Biosci..

